# Exosomes released by granulocytic myeloid-derived suppressor cells attenuate DSS-induced colitis in mice

**DOI:** 10.18632/oncotarget.7324

**Published:** 2016-02-11

**Authors:** Yungang Wang, Jie Tian, Xinyi Tang, Ke Rui, Xinyu Tian, Jie Ma, Bin Ma, Huaxi Xu, Liwei Lu, Shengjun Wang

**Affiliations:** ^1^ Department of Laboratory Medicine, The Affiliated People's Hospital, Jiangsu University, Zhenjiang, China; ^2^ Institute of Laboratory Medicine, Jiangsu Key Laboratory of Laboratory Medicine, Jiangsu University, Zhenjiang, China; ^3^ Department of Pathology, The University of Hong Kong, Hong Kong, China

**Keywords:** inflammatory bowel disease, exosomes, myeloid-derived suppressor cells, inflammation, Immunology and Microbiology Section, Immune response, Immunity

## Abstract

Myeloid-derived suppressor cells (MDSC) have been described in inflammatory bowel disease (IBD), but their role in the disease remains controversial. We sought to define the effect of granulocytic MDSC-derived exosomes (G-MDSC exo) in dextran sulphate sodium (DSS)-induced murine colitis. G-MDSC exo-treated mice showed greater resistance to colitis, as reflected by lower disease activity index, decreased inflammatory cell infiltration damage. There was a decrease in the proportion of Th1 cells and an increase in the proportion of regulatory T cells (Tregs) in mesenteric lymph nodes (MLNs) from G-MDSC exo-treated colitis mice. Moreover, lower serum levels of interferon (IFN)-γ and tumor necrosis factor (TNF)-α were detected in G-MDSC exo-treated colitis mice. Interestingly, inhibition of arginase (Arg)-1 activity in G-MDSC exo partially abrogated the spontaneous improvement of colitis. In addition, G-MDSC exo could suppress CD4^+^ T cell proliferation and IFN-γ secretion *in vitro* and inhibit the delayed-type hypersensitivity (DTH) response, and these abilities were associated with Arg-1 activity. Moreover, G-MDSC exo promoted the expansion of Tregs *in vitro*. Taken together, these results suggest that G-MDSC exo attenuate DSS-induced colitis through inhibiting Th1 cells proliferation and promoting Tregs expansion.

## INTRODUCTION

Inflammatory bowel disease (IBD) is a non-specific, inflammatory, autoimmune disorder with abdominal pain, diarrhea, and bloody stools that may lead to the development of cancer [1]. In humans, IBD includes ulcerative colitis (UC) and Crohn's disease (CD) and is caused by the body's immune response to the normal flora which should normally be tolerable and other factors of host [2]. Studies have shown that Th1 cells and secreted inflammatory factors promoted the development of IBD [3]. Hofseth and colleagues showed that resveratrol protected against DSS induced colitis mouse through up-regulating the silent mating type information regulation-1 (SIRT-1) of immune cells in the colon [4]. However, regulatory T cells (Tregs) play positive roles in maintaining intestinal immune balance [5]. Tregs are decreased in patients with IBD [6], resulting in an imbalance between pro-inflammatory Th1 cells and anti-inflammatory Tregs. The incidence of IBD is on the rise globally. Currently, IBD patients are treated symptomatically [7], and there is an urgent need for effective and curative medical treatments. MDSC are broadly considered as a heterogeneous population of immature myeloid cells that dampen the immune response and accumulate in pathological cases of tumor, inflammation and pathogen infection [8]. MDSC in mice are widely identified as Gr-1 (consisting of Ly6G and Ly6C markers) and CD11b double-positive cells. The two major populations of MDSC can be differentiated by morphologic characteristics and the expression of Gr-1 molecule; CD11b^+^Ly6G^+^Ly6C^low^ cells with granulocyte-like morphology are defined as granulocytic MDSC (G-MDSC), and CD11b^+^Ly6G^−^Ly6C^high^ cells with monocyte-like morphology are defined as monocytic MDSC (M-MDSC) [9]. Both types expand greatly in different tumor models, and the number of G-MDSC is significantly higher than that of M-MDSC. Moreover, the immunosuppressive mechanism of G-MDSC is different from that of M-MDSC [10]. G-MDSC suppress innate and adaptive immune responses through mechanisms involving L-arginine metabolism and reactive oxygen species (ROS) production [11, 12]. These immunosuppressive roles of G-MDSC imply that they can be used to treat autoimmune diseases [13]. In fact, MDSC have been employed to treat collagen-induced arthritis (CIA) and showed a certain effect on reducing severity [14]. Ioannou and colleagues demonstrated that G-MDSC ameliorated experimental autoimmune encephalomyelitis through inhibition of encephalitogenic Th1 and Th17 immune responses [15]. Another study showed that the increased elimination of G-MDSC was due to the extracellular trap (ET) formation driven by the inflammatory milieu of lupus and demonstrate the role of cytokines such as IFN-α, IFN-γ and IL-6 in the induction of ET released by G-MDSC correlated with the production of ROS [16]. However, the exact role of MDSC in IBD pathogenesis is unclear, and there are controversies regarding their immunosuppressive functions in this context [17, 18].

Exosomes (exo) are 30–150-nm phospholipid bilayer-enclosed vesicles that are either released from the parental cell into the extracellular space when multivesicular bodies fuse with the plasma membrane or released directly from the plasma membrane [19]. One study demonstrated that almost all living cells can secrete exo, which are widely present in various biological fluids [20]. It is becoming increasingly clear that exo have specialized functions and play key roles in intercellular signaling, coagulation, and waste management [21]. Exo are also reportedly involved in immune response, apoptosis, angiogenesis, inflammation, and tumor development [22]. Consequently, there is growing interest in their clinical applications for therapy, prognosis, and disease and health biomarkers. From the perspective of disease prevention, exo exhibit more advantages than parental cells [23]. Firstly, exo storage and transport are simple, and they can be used with less cytotoxicity and biohazard. Secondly, the complex molecules on the surface of exo offer potential mechanisms of homing to specific tissues and microenvironments. Lastly, the therapeutic proteins and nucleic acids carried by exo are not easily degraded. Studies have demonstrated that immature dendritic cell-derived exo are a promising subcellular vaccine for autoimmunity, and exo released from iDCs secreting transforming growth factor (TGF)-β1 prevented the development of autoimmune diseases [24].

In this study, G-MDSC were sorted from the spleens of tumor-bearing mice, then G-MDSC exo were isolated from the culture supernatant of G-MDSC by serial centrifugation and purification with an isolation kit. We found that G-MDSC exo could attenuate DSS-induced colitis and restore intestinal immune balance. Subsequently, we confirmed that G-MDSC exo suppress CD4^+^ T cell proliferation and the delayed-type hypersensitivity (DTH) response, and these roles were partially related to arginase (Arg)-1 activity. Moreover, G-MDSC exo could promote Tregs expansion *in vitro*. Collectively, these data indicate that G-MDSC exo have a strong ability to reduce the severity of DSS-induced colitis. Our findings provide a potential immunotherapy for IBD and other autoimmune diseases.

## RESULTS

### Extraction and identification of G-MDSC exo

**Figure 1:**
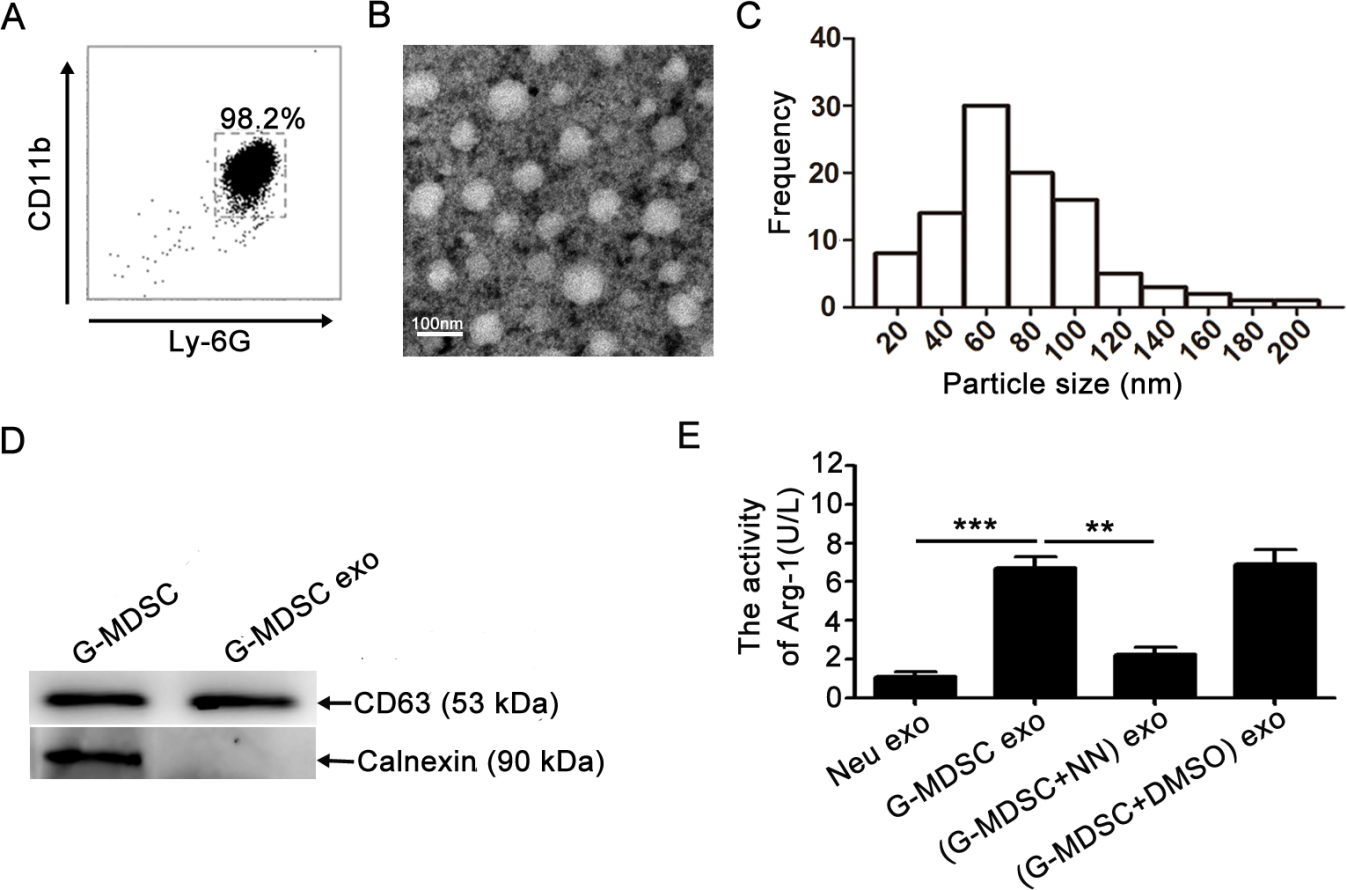
Extraction and identification of G-MDSC exo (**A**) Immunomagnetic beads were used to sort G-MDSC from the spleens of tumor-bearing mice. Ly-6G and CD11b expression levels were analyzed by FCM. (**B**) Representative transmission electron micrograph of G-MDSC exo (magnification, × 80,000; scale bar = 100 nm). (**C**) The particle-size distribution of G-MDSC exo dispersed in fluid. (**D**)CD63 and calnexin expressions were detected by western blot. Data shown in A, B, and D are from one of three independent experiments. (**E**)Exosome Arg-1 activity was detected with an Arg-1 activity assay kit. In the inhibition assay, G-MDSC were treated with the inhibitor nor-NOHA. Data are shown as the mean ± SEM of each group (*n* = 6) pooled from three independent experiments. **p < 0.01, ***p< 0.001, analyzed by ANOVA and *Q* test.

G-MDSC were isolated from the spleen of tumor-bearing mice and analyzed by FCM; cell purity was > 95% (Figure 1A). We could acquire 1 × 10^7^ G-MDSC from one tumor-bearing mouse. G-MDSC were cultured *in vitro*, and culture supernatants were collected after 20 h. Exosomes in the supernatants were purified by the method of differential centrifugation followed by use of exosomes extraction kits. G-MDSC exo were observed by transmission electron microscopy, which revealed that most of the exosomes displayed closed round vesicles with diameters of 30–150 nm (Figure 1B and 1C). Further characterization indicated that G-MDSC exo had a similar level of exo-associated CD63 (Figure 1D). In contrast, calnexin was not detected in the purified G-MDSC exo preparations (Figure 1D), indicating that G-MDSC exo were free from contamination with non-exo membrane proteins. We could acquire 3–5 μg G-MDSC exo from 1 × 10^6^ G-MDSC.

Arg-1 is one of the most important molecules that plays a critical role in the immunosuppressive function of G-MDSC. To determine whether G-MDSC exo contain Arg-1 activity, the Arg-1 activity of G-MDSC exo was detected with an assay kit according to the manufacturer's instructions. The results showed Arg-1 activity in G-MDSC exo (Figure 1E). Additionally, the specific arginase inhibitor N^ω^-hydroxy-nor-Arginine (nor-NOHA, also NN) was added to the G-MDSC culture system, and this type of exo was isolated and termed (G-MDSC+NN) exo; they showed significant decrease of Arg-1 activity (Figure 1E).

### G-MDSC exo attenuate DSS-induced experimental colitis in mice

**Figure 2:**
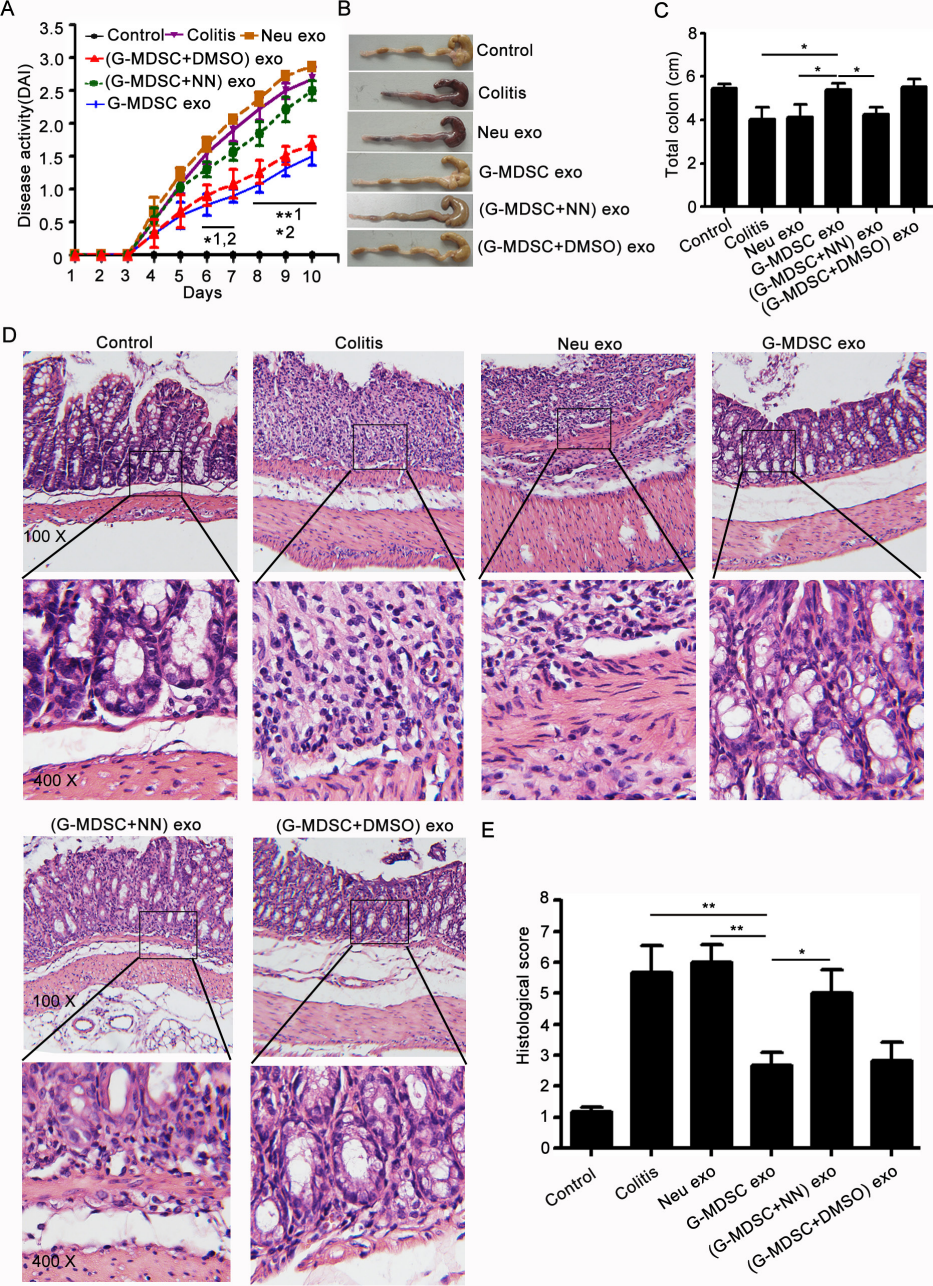
G-MDSC exo attenuate the severity of DSS-induced colitis Murine experimental colitis was induced in C57BL/6 mice (*n* = 10/group) by providing 2.5% DSS solution in their drinking water for 10 days. Mice received i.p. injections with exosomes (30 μg/mouse/injection) on days 2, 4, and 6. (**A**) Disease activity was determined daily as described in the methods. Mice were sacrificed on day 8 by eye bloodletting followed by cervical dislocation. Colon sections obtained from mice were analyzed for their outward appearance (**B**), length (**C**), degree of inflammation (**D**) and H & E histologic scoring. (**E**) All data are presented as the mean ± SEM from one of three independent experiments. *p < 0.05, **p < 0.01; 1, G-MDSC exo-treated colitis mice versus colitis mice; 2, G-MDSC exo-treated colitis mice versus (G-MDSC+NN) exo-treated colitis mice, analysis with ANOVA and *Q* test. The mice of the control group had no DSS treatment. The mice of the colitis group had DSS treatment, while no exo treatment. All other experimental groups had DSS and different exos treatment.

To test whether G-MDSC exo can ameliorate colitis, C57BL/6 mice were injected i.p with G-MDSC exo (30 μg/mouse/injection) on days 2, 4, and 6 after colitis induction. Multiple observations collectively indicated that G-MDSC exo-treated mice were considerably less susceptible to DSS-induced colitis compared to other groups. Firstly, the DAI of G-MDSC exo-treated colitis mice was lower than in colitis mice or (G-MDSC+NN) exo-treated colitis mice on day 4 after the initiation of DSS treatment. Moreover, this difference gradually increased over time (Figure 2A). Secondly, DSS treatment caused severe bloody stools that persisted until the sacrifice of colitis mice or (G-MDSC+NN) exo-treated colitis mice. This change was not observed in G-MDSC exo-treated colitis mice (Figure 2B). Thirdly, we measured the length of colon from mice in different groups. Results showed that the length of colon in colitis mice or (G-MDSC+NN) exo-treated colitis mice is shorter than G-MDSC exo-treated colitis mice (Figure 2C). Finally, histologic examination of colonic sections revealed complete disruption of the colonic architecture in colitis mice, whereas G-MDSC exo-treated colitis mice retained intact colonic architecture, and the severity of disease in (G-MDSC+NN) exo-treated colitis mice was greater than G-MDSC exo-treated colitis mice (Figure 2D). Consistent with histologic observations, the histologic scoring of G-MDSC exo-treated colitis mice was significantly lower than colitis mice, G-MDSC exo-treated colitis mice or (G-MDSC+NN) exo-treated colitis mice (Figure 2E). Taken together, these results strongly support the hypothesis that G-MDSC exo could attenuate DSS-induced murine experimental colitis and suggest that Arg-1 plays an important role in this process.

### G-MDSC exo prevent Th1 cell development and promote Tregs expansion in murine experimental colitis

**Figure 3:**
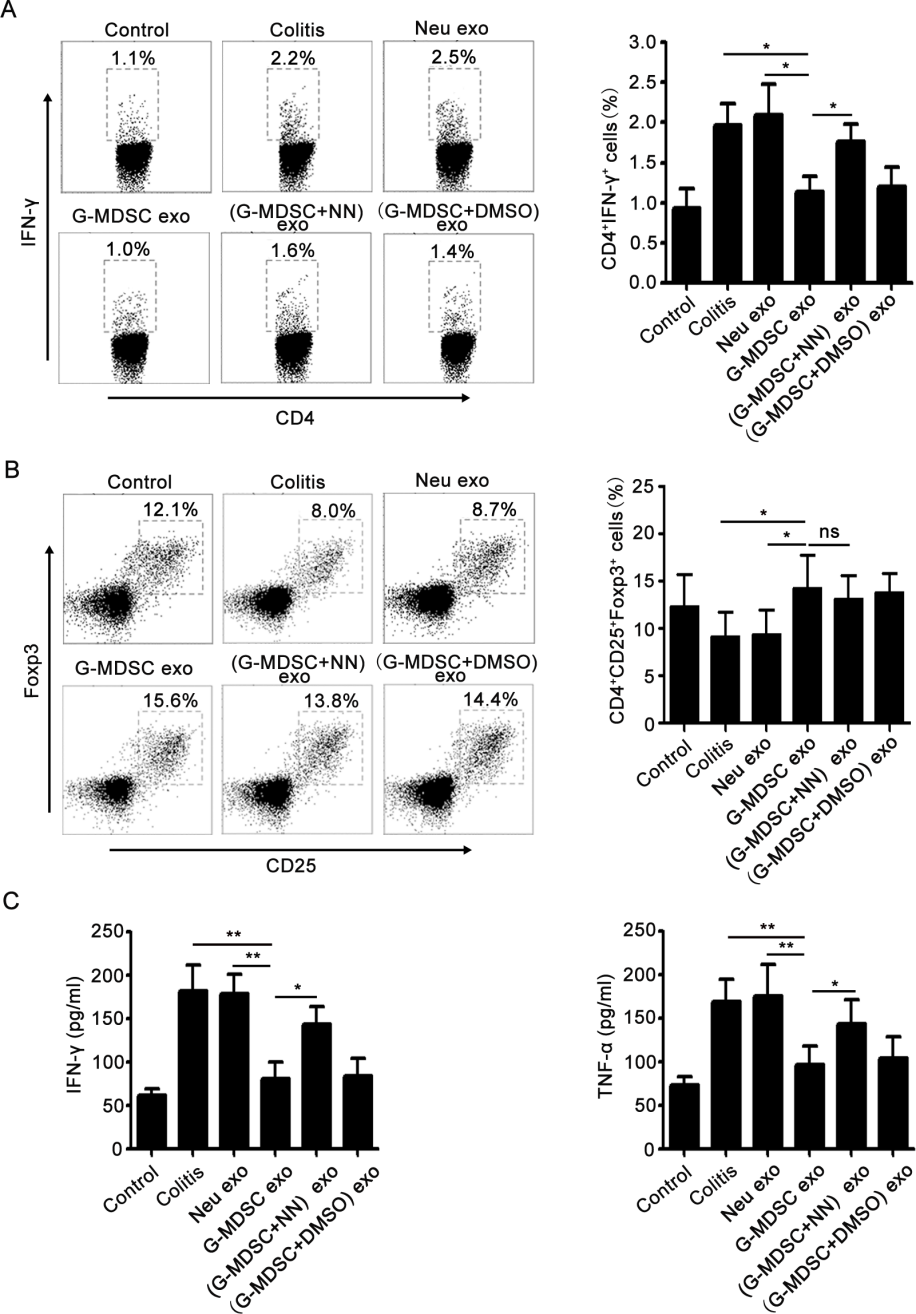
G-MDSC exo prevent the Th1 cell response and expand Tregs in murine experimental colitis Mice were sacrificed, and lymphocytes from the MLNs of different groups of DSS-induced colitis mice were isolated on day 8. (**A**) For Th1 cell detection, lymphocytes were stimulated for 5 h with 1 μg/ml ionomycin and 50 ng/ml phorbol myristate acetate in the presence of 2 mg/ml monensin at 37°C and stained with anti-mouse CD4 and IFN-γ mAbs according to the manufacturer's protocols. The percentage of Th1 cells was analyzed by FCM (left), and the statistical analysis results are shown (right). (**B**) For the detection of Tregs, lymphocytes were stained with anti-mouse CD4, CD25, and Foxp3 mAbs as described in the methods. The percentage of Tregs was analyzed by FCM (left), and the statistical analysis results are shown (right). (**C**) Serum cytokine concentrations in different groups after 8 days of DSS treatment. Data are shown as the mean ± SEM of each group (*n* = 10) pooled from three independent experiments. *p < 0.05, **p < 0.01, ns: not significant, analyzed by ANOVA and *Q* test.

Th1 cells and IFN-γ are crucial for the pathogenesis of IBD, and TNF-α also participates in IBD pathogenesis [25]. However, Tregs could maintain the intestinal immune balance and play a protective role in IBD [5]. To understand the mechanisms underlying the action of G-MDSC exo against colitis, the percentages of Tregs and Th1 cells in MLNs were analyzed by FCM, and the serum levels of IFN-γ and TNF-α were determined by ELISA. As shown in Figure 3A, following treatment with G-MDSC exo, the percentage of Th1 cells was significantly lower than that in colitis, Neu exo-, or (G-MDSC+NN) exo-treated colitis mice. However, the percentage of Tregs in G-MDSC exo-treated colitis mice was significantly higher than that in colitis or Neu exo-treated colitis mice (Figure 3B). Moreover, markedly lower levels of IFN-γ and TNF-α were detected in the serum of G-MDSC exo-treated colitis mice compared with that in other groups of colitis mice (Figure 3C). Hence, our data indicate that treatment with G-MDSC exo inhibits Th1 cell responses but enhances Tregs, which contributes to the inhibition of colitis.

### G-MDSC exo suppress CD4^+^ T cell proliferation and IFN-γ secretion *in vitro*

**Figure 4:**
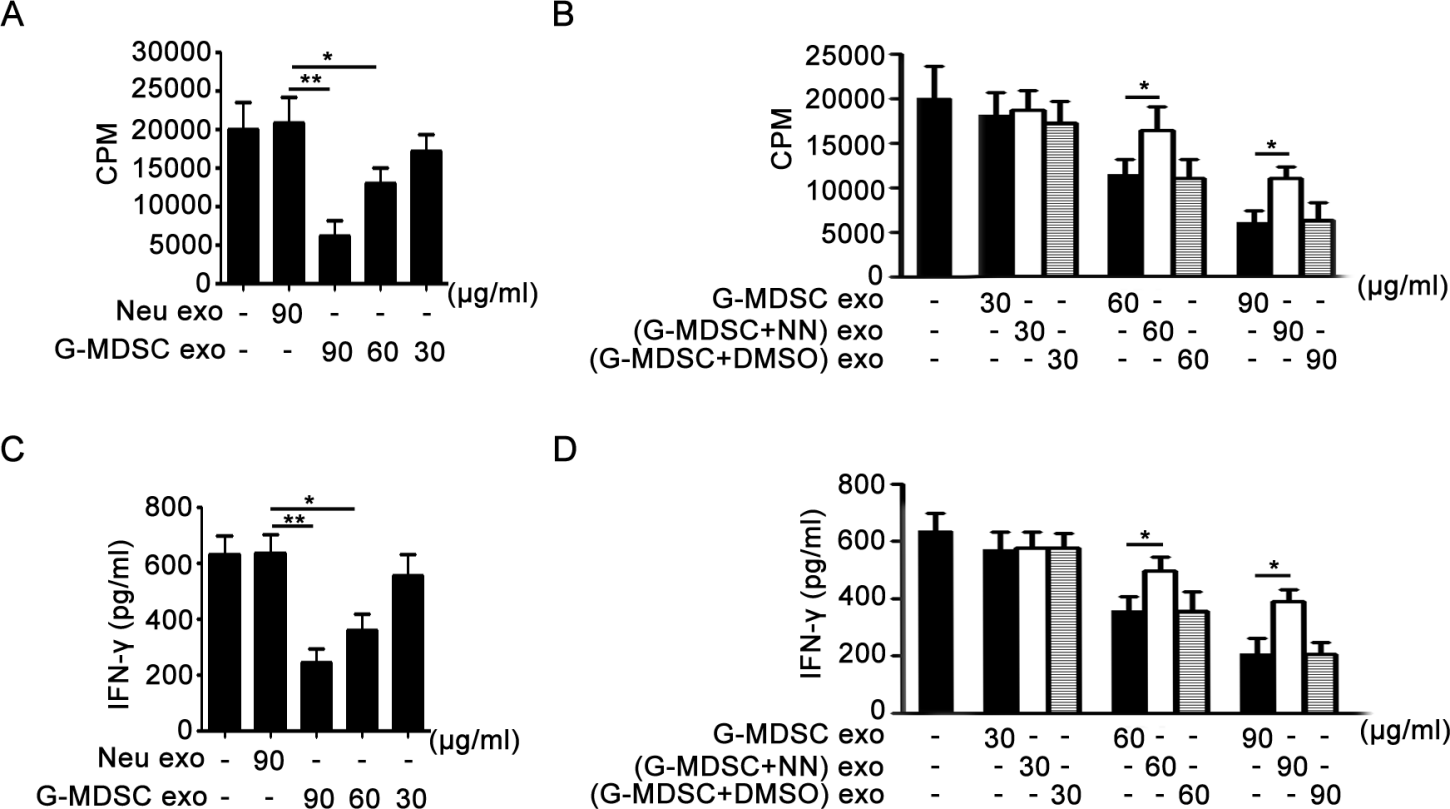
G-MDSC exo suppress CD4^+^ T cell proliferation and IFN-γ secretion correlating with Arg-1 activity *in vitro* (**A** and **B**). G-MDSC exo suppress CD4^+^ T cell proliferation through Arg-1 activity. CD4^+^ T cells were treated with G-MDSC exo (A) or/and (G-MDSC+NN) exo (B) at different concentrations for 72 h. The cultivation system was stimulated with anti-CD3 mAb (2 μg/ml) and anti-CD28 mAb (2 μg/ml). Cell proliferation was measured by [^3^H]-thymidine incorporation. (**C** and **D**). G-MDSC exo suppress IFN-γ secretion through Arg-1 activity. The levels of IFN-γ in culture supernatants from the CD4^+^ T cell proliferation system were detected using sandwich ELISAs. Data are shown as the mean ± SEM of each group (*n* = 6) pooled from three independent experiments. *p < 0.05, **p < 0.01, analyzed by ANOVA and *Q* test.

Based on the immunosuppressive effect of G-MDSC exo on Th1 cells in DSS-induced murine colitis, we examined the role of G-MDSC exo in CD4^+^ T cell proliferation and IFN-γ secretion from CD4^+^ T cells *in vitro*. Moreover, we observed the role of Arg-1 activity in the immunosuppression of G-MDSC exo. In the presence of anti-CD3 and anti-CD28 mAbs, CD4^+^ T cells isolated from the spleen of male wild-type C57BL/6 mice were cultivated in the presence of varying concentrations of G-MDSC exo or equimolar concentrations of Neu exo for 3 days. The CPM values in G-MDSC exo-treated groups were lower than in the untreated or Neu exo-treated groups, and the degree of CPM value reduction in G-MDSC exo-treated groups correlated with the amount of G-MDSC exo (Figure 4A). However, the CPM values in medium and high concentrations (G-MDSC+NN) exo-treated groups were significantly lower than those of G-MDSC exo-treated groups (Figure 4B). Notably, the changes of IFN-γ levels in culture supernatants were consistent with the changes of CD4^+^ T cell proliferation in different groups (Figure 4C and 4D). These results confirmed that G-MDSC exo suppressed CD4^+^ T cell proliferation and IFN-γ secretion in a dose-dependent manner, and this inhibitory effect correlated with Arg-1 activity.

### G-MDSC exo suppress DTH response

**Figure 5:**
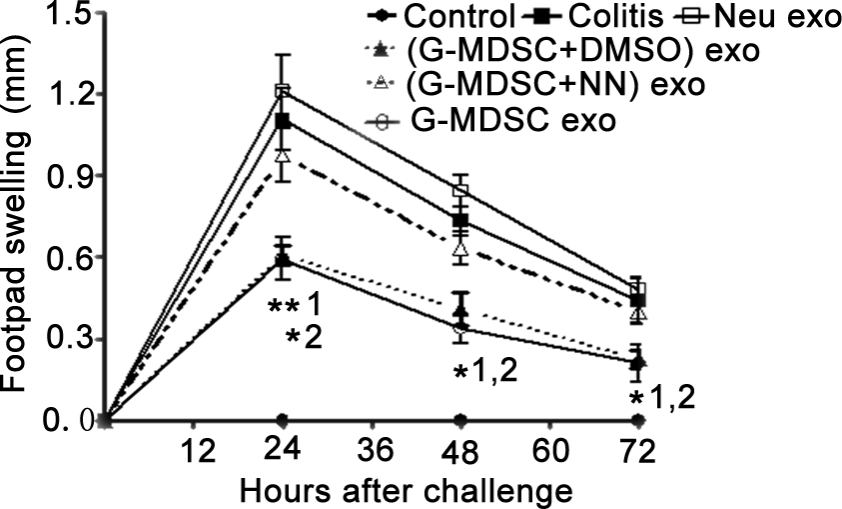
G-MDSC exo suppress the DTH reaction in an Arg-1-dependent manner OVA-sensitized mice were challenged with OVA in the footpads, and paw edema was measured at 24, 48, and 72 h after the challenge. Footpad swelling was calculated according to the formula described in the methods. Data are shown as the mean ± SEM of each group (*n* = 6) pooled from three independent experiments. *p < 0.05, **p < 0.01; 1, G-MDSC exo-treated DTH mice versus DTH mice; 2, G-MDSC exo-treated DTH mice versus (G-MDSC+NN) exo-treated DTH mice, analyzed by ANOVA and *Q* test.

The DTH reaction mainly correlated with Th1 cells. To further confirm the role of G-MDSC exo in suppressing the response of CD4^+^ T cells *in vivo*, we investigated the role of G-MDSC exo in DTH. The DTH model involved an OVA challenge in mouse footpads, and footpad swelling was measured at a given time after the challenge. The DTH response was significantly suppressed in mice treated with G-MDSC exo before the OVA challenge (Figure 5). Moreover, this effect was weakened in (G-MDSC+NN) exo-treated mice (Figure 5).

### G-MDSC exo promote TGF-β-induced Tregs generation from CD4^+^ T cells *in vitro*

**Figure 6:**
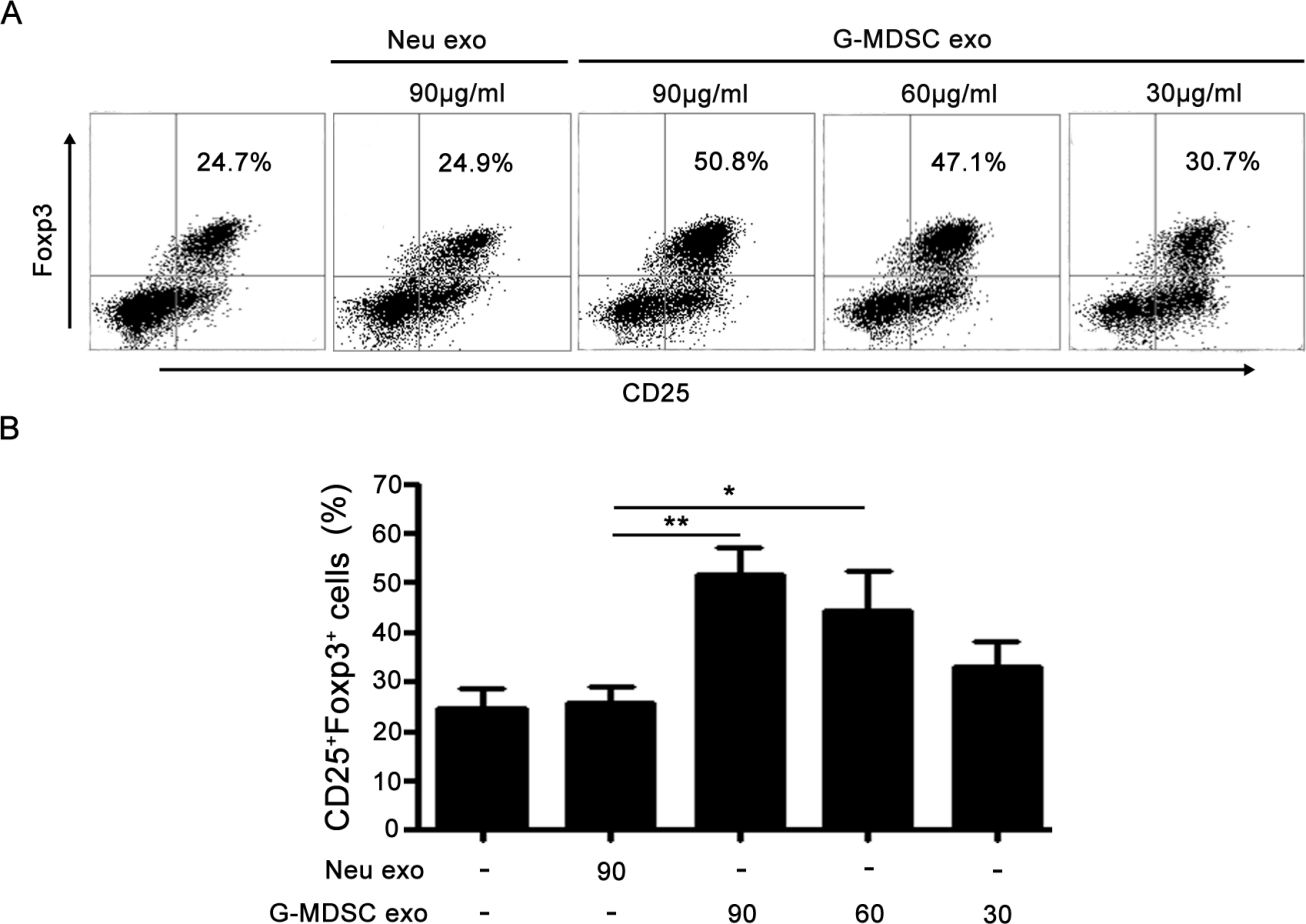
G-MDSC exo promote TGF-β induced Tregs generation from CD4^+^ T cells 1.5 × 10^6^/ml CD4^+^ T cells were isolated from the spleen of C57BL/6 mice and cultured for 72 h with anti-CD3 (2 μg/ml), anti-CD28 (2 μg/ml) Ab, and TGF-β (5 ng/ml) in the presence or absence of exo isolated from G-MDSC or neutrophils. The cells were stained with anti-mouse CD4, CD25, and Foxp3 mAbs. The cells were gated first on living lymphocytes and then CD4^+^ T cells. Tregs percentages were analyzed by FCM. Representative dot plots of Tregs (**A**) and the percentages of Tregs from different treatments (**B**). Data are shown as the mean ± SEM of each group (*n* = 6) pooled from three independent experiments, analyzed by ANOVA and *Q* test. *P < 0.05; **P < 0.01.

Given the effect of G-MDSC exo on Tregs expansion in murine IBD and previous reports indicated that MDSC promoted Tregs expansion [26, 27]. We assumed that G-MDSC exo might promote the generation of Tregs from CD4^+^ T cells. To test this hypothesis, we evaluated the influence of G-MDSC exo on the expansion of Tregs from CD4^+^ T cells. CD4^+^ T cells isolated from C57BL/6 mice splenocytes were cultured for 72 h in the presence or absence of exo isolated from G-MDSC or neutrophils. Tregs percentages were determined by FCM. As shown in Figure 6A and 6B, a significant dose-dependent increase in Tregs percentage was observed in the presence of G-MDSC exo.

## DISCUSSION

Exosomes are highly abundant in biologic fluids where they transfer information between cells. A report showed that MDSC isolated from BALB/c mice carrying 4T1 mammary carcinomas shed exosomes that contain multiple bioactive components derived from many subcellular compartments and are associated with diverse functions [22]. In this study, we found G-MDSC exo were bilayered vesicles as having diameters in the 30–150-nm range by transmission electron micrographs, which is consistent with previously described exosomes [28]. It is noteworthy that G-MDSC exo possess Arg-1 activity, which plays a critical role in the immunosuppressive function of G-MDSC. Overall, these results indicate that G-MDSC exo share some biological functions with their parental cells.

Although studies have attempted to utilize MDSC to suppress the autoimmune response and limit tissue injury in the context of EAE [15, 29] and CIA [14, 30], and get beneficial results. However, the roles of MDSC in IBD pathogenesis remain unclear, and the reports of immunosuppressive functions of MDSC in IBD are fairly controversial [17, 18]. Haile et al. showed that MDSC frequency dramatically increased during intestinal inflammation in a CD8^+^ T-cell-mediated mouse model of IBD and suppressed IFN-γ secretion from T cells, and transferred MDSC decreased intestinal inflammation [31]. Recruited myeloid cells may acquire antigen-presenting functions after undergoing a series of phenotypic and functional changes and stimulate cytokine production by effector Th1/Th17 cells in chronic colitis [32]. Moreover, inflammatory monocytes recruited in the lamina propria of colon may differentiate into proinflammatory dendritic cells [33]. However, these cells express inducible nitric oxide synthase and Arg-1, suggesting a more activated state resulting in an inability to suppress activated/effector Th1/Th17 cells [34]. Considering the exact role of MDSC in IBD pathogenesis is unclear, and the clear immunosuppression and rich content of G-MDSC in tumor-bearing mice, we used G-MDSC exo to inhibit the development of DSS-induced colitis. The application of G-MDSC exo attenuated DSS-induced colitis, which was accompanied by a decrease in the number of Th1 cells and an increase in the number of Tregs from MLNs. Moreover, G-MDSC exo reduced the serum levels of IFN-γ and TNF-α in DSS-induced colitis mice. Inhibition of Arg-1 activity with nor-NOHA partly attenuated the improvement of DSS-induced colitis by G-MDSC exo. G-MDSC play the role of immune suppression by the production of ROS, Arg-1 and generation of NO [10]. Peroxynitrite which formed by the cooperative activity of ROS with NO is another factor that inhibits effector T cells [35]. Peroxynitrite leads to the nitration of tyrosines in the T-cell receptor (TCR). This reaction damages the conformational flexibility of TCR complex, and inhibits its binding with peptide-loaded MHC and leads to the unresponsiveness of T cells to antigen-specific stimulation [8, 15, 36, 37]. The suppressive activity of Arg-1 is based on its role in the hepatic urea cycle, metabolizing L-arginine to L-ornithine. Expression of Arg-1 has been reported to decrease CD3ξ-chain biosynthesis and down-regulate TCR on cell surface and leads to the unresponsiveness of T cells without MHC II restriction and in an antigen-non-specific manner [38]. In our study, G-MDSC exo mediate their suppressive activity on autoimmune IBD mice through preventing Th1 cell reaction which depended on Arg-1 activity. These regulation on T effector immune responses without MHC II restriction and in an antigen-non-specific manner. Collectively, these results showed that G-MDSC exo can inhibit DSS-induced colitis, and this was associated with Arg-1 activity-mediated suppression of Th1 cells and Tregs expansion.

To demonstrate the immunosuppressive effect of G-MDSC exo, we observed the roles of G-MDSC exo on CD4^+^ T cell proliferation and Tregs expansion *in vitro*. We found that G-MDSC exo could inhibit CD4^+^ T cell proliferation and IFN-γ secretion, and these effects correlated with Arg-1 activity. Moreover, these effects were further confirmed by our observation that G-MDSC exo alleviated the DTH reaction *in vivo*. In addition, we found G-MDSC exo promoted Tregs expansion from CD4^+^ T cells in the presence of TGF-β, although the exact reasons need further study. One certainty is that MDSC promote Tregs expansion [26, 27, 39]. This is consistent with the theory that exosomes have some properties in common with their parent cells. These experimental results suggested G-MDSC exo have strong immunosuppressive activity *in vitro* and are consistent with the roles of G-MDSC exo in DSS-induced colitis.

In conclusion, we successfully isolated and identified G-MDSC exo, providing an experimental basis for further studies. Our findings suggest that G-MDSC exo attenuate DSS-induced murine colitis by suppressing pathogenic Th1 cells and inducing Tregs differentiation and the protective effect correlated with arginase activity in MDSC exo. Moreover, these immunosuppressive effects of G-MDSC exo were confirmed *in vitro*. In conclusion, our work provides a new way in the development of an effective intervention of IBD and other autoimmune diseases.

## MATERIALS AND METHODS

### Antibodies and reagents

CD4^+^ T cells, G-MDSC isolation kits, and phycoerythrin (PE)-conjugated anti-mouse interferon (IFN)-γ were from Miltenyi Biotec (Bergisch Gladbach, Germany). PE, fluorescein isothiocyanate (FITC), PE-Cy5-conjugated anti-mouse CD4 mAb (L3T4), PE-conjugated anti-mouse Foxp3 mAb (FJK-16s), and Cy5.5-conjugated anti-mouse CD25 mAb (PC61.5) were from eBioscience (San Diego, CA, USA). PE-conjugated anti-mouse Ly-6G mAb (RB6–8C5), CD11b mAb (M1/70), anti-CD3 mAb, and anti-CD28 mAb were from Biolegend (San Diego, CA, USA). Mouse mAbs against CD63 (Y-18) and calnexin (C8. B6) were from Abcam (Cambridge, UK). IFN-γ and tumor necrosis factor (TNF)-α enzyme-linked immunosorbent assay (ELISA) kits were from MultiSciences (Shanghai, China). QuantiChrom^™^ arginase assay kits were from Bioassays (Hayward, CA, USA). ExoQuick-TCTM Exosomes were from SBI (Mountain View, CA, USA). Recombinant mouse TGF-β was from PeproTech (Rocky Hill, NJ, USA). MicroBCA protein assay kits were from Beijing ComWin Biotech (Beijing, China). Ovalbumin (OVA) and complete Freund's adjuvant (CFA) were from Sigma (St. Louis, MO, USA). N^ω^-hydroxy-nor-Arginine was from Cayman (Ann Arbor, MI, USA).

### Mice

Male C57BL/6 mice (6–8 weeks old, weighing 18–22 g) were from the Animal Research Center of Jiangsu University (Zhenjiang, China) and were housed in a specific pathogen-free facility. The experimental protocols were approved by the Jiangsu University Animal Ethics and Experimentation Committee.

### G-MDSC isolation

Tumor-bearing mice were established with the Lewis lung adenocarcinoma cell line according to a method previously used in our laboratory [9, 40]. When tumors were greater than 2 cm in diameter, G-MDSC were harvested from the spleen with G-MDSC isolation kits according to the manufacturer's instructions, and their purity was assessed by measuring the expression of Ly-6G and CD11b with flow cytometry (FCM).

### G-MDSC exo purification

To make preparations for large-scale exosomes purification, isolated G-MDSC were cultivated in an incubator at 37°C and 5% CO_2_ for 20 h in G-MDSC-conditioned medium (R1640 with 10% fetal bovine serum that had been ultracentrifuged at 100,000 g for 16 h at 4°C). The culture supernatant of G-MDSC was harvested after 20 h. Exosomes were purified from the supernatant by differential centrifugation and ultrafiltration membrane technology followed by the use of an exosomes extraction kit. In brief, cells and cellular debris were removed by sequential centrifugation at 500 g for 10 min, 1000 g for 30 min, and 10000 g for 30 min. The concentrated supernatant was acquired by an ultrafiltration membrane with a molecular weight cut-off ranging from 2 to 100 kDa. The supernatant was passed through a 0.22-μm microcentrifuge filter. The filtrate was mixed with exosomes isolation reagent (v/v = 5:1) and incubated for 16 h at 4°C. Finally, the mixture was centrifuged at 1000 g for 30 min, and the precipitate was G-MDSC exo. Exosomes were dissolved in phosphate-buffered saline (PBS) and stored at −80°C. The protein contents of G-MDSC exo were quantified using a microBCA protein assay kit. In this study, we also prepared neutrophil-derived exo (Neu exo), which served as control for G-MDSC exo.

In the purification process of (G-MDSC+NN) exo in which the Arg-1 activity is inhibited, G-MDSC used to extract exosomes were cultured in the presence of 200 μM nor-NOHA, which is a potent inhibitor of arginase. Because nor-NOHA is dissolved in dimethyl sulfoxide (DMSO), we prepared (G-MDSC+DMSO) exo as solvent to control for the influence of DMSO on the experiment.

### G-MDSC exo transmission microscopic examination

Purified G-MDSC exo suspended in 2% glutaraldehyde in PBS were loaded on a formvar-coated grid and negatively stained with 3% (w/v) aqueous phosphotungstic acid for 1 min. Electron micrographs were observed by transmission electron microscopy (Tecnai-12; Philips, Amsterdam, Netherlands).

### Western blot analysis

G-MDSC and G-MDSC exo were lysed in radioimmunoprecipitation (RIPA) buffer, and lysates were separated by 12% sodium dodecyl sulfate-polyacrylamide gel electrophoresis (SDS-PAGE) and subsequently electrotransferred onto Immobilon polyvinylidene membranes (Bio-Rad, Hercules, CA, USA), and probed with mouse mAbs against CD63 or calnexin, and HRP conjugated anti-mouse IgG followed by chemiluminescent detection (Champion Chemical, Whittier, CA, USA).

### Detection of arginase activity

Arg-1 activity contents in the lysates from exosomes or G-MDSC were measured by Arg-1 activity assay according to the manufacturer's protocols. Briefly, 2 × 10^6^ G-MDSC or exosomes isolated from 2 × 10^6^ G-MDSC or neutrophil were lysed in 50 μl RIPA buffer, collected the lysate by centrifugation at 14000 g for 10 min at 4^°^C, and added ddH_2_O to 100 μl. Arginase activity was detected with the QuantiChrom Argianse Assay kit (BioAssay systems, Hayward, CA) following the manufacturer's instructions.

### DSS-induced experimental colitis in mice

DSS-induced murine experimental colitis was established as described previously with minor modification [41]. Briefly, male C57BL/6 mice were treated with 2.5% DSS in drinking water for 10 days to induce colon injury and colitis, and these mice were treated with PBS or exosomes (30 μg/mouse/i.p) on days 2, 4, and 6 after DSS drinking. Normal control mice were given normal drinking water and were treated with PBS. Weight loss, rectal bleeding and diarrhea were monitored daily for 10 days and graded separately on scales of 0–4. The average of the three values constitutes the disease activity index (DAI). Mice were sacrificed on day 8 by eye bloodletting followed by cervical dislocation. Colons were mechanically isolated, cleaned, and measured in length. After removing caecum and adipose tissue, the colons were fixed in 10% formalin solution, paraffin-embedded, sections, stained with hematoxylin and eosin (H & E), and examined under a light microscope. Histological scoring was done as described previously [42].

### FCM analysis

On day 8, mice were sacrificed by eye bloodletting followed by cervical dislocation. MLNs were isolated and grinded in 2 ml PBS. The cell suspensions were filtered through 70 μm cell strainers. The lymphocytes were collected by centrifugation at 500 g for 5 min at 4^°^C. For Tregs detection, lymphocytes were stained with anti-mouse CD4 and CD25 and Foxp3 mAbs, and defined the proportion of CD25^+^Foxp3^+^ lymphocytes gated in CD4^+^ lymphocytes as described previously [43]. For the detection of Th1 cells, single-cell suspensions were stimulated with 50 ng/ml phorbol myristate acetate, 1 μg/ml ionomycin, and 2 μg/ml monensin. After 5 h, cells were stained with anti-CD3 and anti-CD4 mAbs, fixed, permeabilized, and stained with anti-IFN-γ mAb according to the intracellular staining kit (Invitrogen, Carlsbad, CA, USA) instructions. Defined the proportion of CD4^+^IFN-γ^+^ lymphocytes gated in CD3^+^ lymphocytes.

### ELISA

On day 8, mice were sacrificed by eye bloodletting followed by cervical dislocation. The eyeball blood was collected into 1.5 ml EP tubes without anticoagulant and the serum was isolated by centrifugation for 5 min at 3000 rpm. IFN-γ and TNF-α contents in the serum from different groups of mice were detected with the Mouse IFN-γ or TNF-α SunnyELISA Assay kit (Multi sciences, Hangzhou, China) following the manufacturer's instructions.

### T-cell proliferation assay

For *in vitro* experiments, CD4^+^ T cells were isolated from the splenocytes of wild-type C57BL/6 mice with a CD4^+^ T cell isolation kit as previously described [44]. Briefly, 1 × 10^6^/ml cells were stimulated with anti-CD3 (5 μg/ml) and anti-CD28 (1 μg/ml) mAbs in triplicate in round-bottom 96 wells. Different doses of each type of exosomes were added into the wells. Cells were cultured in a humidified 5% CO_2_ atmosphere at 37°C for 72 h, and [^3^H]-thymidine (1 μCi/well; Pharmacia, Uppsala, Sweden) was added for the last 16 h. The counts per minute (CPM) values of various wells were detected with an LS6500 multi-purpose scintillation counter (Beckman Coulter, Brea, CA, USA).

### Induction of CD4^+^CD25^+^Foxp3^+^ Tregs

CD4^+^ T cells isolated from C57BL/6 mice splenocytes were cultured with anti-CD3 and anti-CD28 mAbs in the presence or absence of 5 ng/ml TGF-β in a 24-well plate for 72 h in complete RPMI medium (1.5 × 10^6^ cells/well) with or without exo isolated from G-MDSC or neutrophils. The percentages of CD25^+^Foxp3^+^ cells in CD4^+^ T cells were determined by FCM after 72 h.

### Induction of DTH

DTH was induced in mice by challenging the footpad of previously sensitized mice with OVA as described previously [45]. In brief, C57BL/6 mice were sensitized by intradermal injections of 1 mg/ml CFA-emulsified OVA peptide (grade V) in a final volume of 200 μl at the tail base and back. Seven days after sensitization, each mouse was challenged by footpad injection of 20 mg/ml PBS-dissolved OVA peptide (grade II) in a final volume of 30 μl. Another rear paw was injected with a comparable volume of PBS as a control. Footpad thickness was measured using a vernier caliper (Mitutoyo Corp, Tokyo, Japan) at a given time after the challenge. The magnitude of the DTH response was determined as follows: DTH units=(footpad thickness of OVA injection [mm])-(footpad thickness of PBS injection [mm]). For DTH treatment, mice were intraperitoneally (i.p.) injected with different exosomes (30 μg/mouse/injection) on days 2, 4, and 6 after sensitization.

### Statistical analysis

The statistical significance of differences between groups was analyzed by ANOVA and *Q* test using the SPSS version 16.0 for Windows (SPSS Inc, Chicago, IL, USA). The data are presented as the mean ± SEM from at least three independent experiments. A *p*-value < 0.05 was considered statistically as significant.
